# Toxicity of Potential Fungal Defense Proteins towards the Fungivorous Nematodes Aphelenchus avenae and Bursaphelenchus okinawaensis

**DOI:** 10.1128/AEM.02051-18

**Published:** 2018-11-15

**Authors:** Annageldi Tayyrov, Stefanie S. Schmieder, Silvia Bleuler-Martinez, David F. Plaza, Markus Künzler

**Affiliations:** a Institute of Microbiology, Department of Biology, ETH Zürich, Zürich, Switzerland; University of Bayreuth

**Keywords:** mycophagy, lectin, avidin, filamentous fungus, Ashbya gossypii, nematotoxicity

## Abstract

Resistance of fungi to predation is thought to be mediated by toxic metabolites and proteins. Many of these fungal defense effectors are highly abundant in the fruiting body and not produced in the vegetative mycelium. The defense function of fruiting body-specific proteins, however, including cytoplasmically localized lectins and antinutritional proteins such as biotin-binding proteins, is mainly based on toxicity assays using bacteria as a heterologous expression system, with bacterivorous/omnivorous model organisms as predators. Here, we present an ecologically more relevant experimental setup to assess the toxicity of potential fungal defense proteins towards the fungivorous, stylet-feeding nematodes Aphelenchus avenae and Bursaphelenchus okinawaensis. As a heterologous expression host, we exploited the filamentous fungus Ashbya gossypii. Using this new system, we assessed the toxicity of six previously characterized, cytoplasmically localized, potential defense proteins from fruiting bodies of different fungal phyla against the two fungivorous nematodes. We found that all of the tested proteins were toxic against both nematodes, albeit to various degrees. The toxicity of these proteins against both fungivorous and bacterivorous nematodes suggests that their targets have been conserved between the different feeding groups of nematodes and that bacterivorous nematodes are valid model organisms to assess the nematotoxicity of potential fungal defense proteins.

**IMPORTANCE** Our results support the hypothesis that cytoplasmic proteins abundant in fungal fruiting bodies are involved in fungal resistance against predation. The toxicity of these proteins toward stylet-feeding nematodes, which are also capable of feeding on plants, and the abundance of these proteins in edible mushrooms, may open possible avenues for biological crop protection against parasitic nematodes, e.g., by expression of these proteins in crops.

## Abstract (precis)

Our results support the hypothesis that cytoplasmic proteins abundant in fungal fruiting bodies are involved in fungal resistance against predation. The toxicity of these proteins toward stylet-feeding nematodes, which are also capable of feeding on plants, and the abundance of these proteins in edible mushrooms, may open possible avenues for biological crop protection against parasitic nematodes, e.g., by expression of these proteins in crops.

## INTRODUCTION

The fungal fruiting body is a relatively short-lived structure that, as a nutrient source, attracts many different predators due to its high and readily available nutrient content (1–6). Its importance to the organism as a sexual reproductive organ is reflected by the plethora of toxic molecules that are constitutively and specifically produced in this tissue at relatively high levels. These molecules have been discussed to reduce the negative impact of predators on the reproductive potential of the organism (7–9) by deterrence (10–15). While the role of many secondary metabolites in defense has been established over the years, the contribution of fruiting body-specific proteins such as lectins or biotin-binding proteins to defense remains still elusive. Similarly to that of secondary metabolites (16, 17), the biosynthesis of these proteins is subject to spatiotemporal regulation during fungal fruiting body development (18, 19).

Lectins are defined as proteins possessing at least one noncatalytic domain that binds reversibly to a specific monosaccharide or oligosaccharide (20). They act as recognition and effector molecules in the innate immune response of several phyla, including invertebrates, mammals, and plants (21, 22). In fungi, lectins are abundant in fruiting bodies and sclerotia (19, 23–25). Their cytoplasmic expression and the absence of cytoplasmic ligands, as well as the lack of developmental phenotypes upon downregulation, argue against an endogenous function, e.g., in fungal development (23, 26, 27). Recently, we tested the toxicity of different bacterially produced fungal fruiting body lectins for their toxicity against invertebrate and protozoan model organisms, including Caenorhabditis elegans, an Acanthamoeba sp., and Aedes aegypti (28). Their toxicity toward these bactivorous and omnivorous organisms suggests that fruiting body lectins may mediate a constitutive protein-based resistance of the fruiting body against predators (28).

Biotin-binding proteins are another class of putative defense molecules expressed in fruiting bodies. The synthesis of biotin is restricted to plants and some microorganisms, making biotin an essential vitamin for most other organisms, including herbivores and fungivores. Biotin-binding proteins are expressed by many different species. They are characterized by a very strong noncovalent binding to biotin and have been implicated as antimicrobial host defense factors that create a “biotin-free zone” (29–32). The cytoplasmic biotin-binding proteins of the basidiomycete Pleurotus cornucopiae, tamavidin 1 (Tam1) and 2 (Tam2), were shown to be toxic to C. elegans, an Acanthamoeba sp., and Drosophila melanogaster (33).

The contribution of individual proteins to fungal resistance toward ecologically relevant fungivores has hardly been investigated in the past. To date, most studies determined the toxicity of individual heterologously expressed proteins to model organisms (18, 28, 33–35). In contrast, for this study, we assessed the potentials of different protein classes as resistance molecules against two fungivorous nematode species. Nematodes are one of the most abundant organisms in the soil ecosystem, and many of them include fungi in their diets or feed exclusively on fungi (36). Thus, nematodes represent an ecologically relevant phylum of fungal predators. Due to their feeding mechanism, i.e., piercing the hyphal cell wall with a stylet, fungivorous nematodes can bypass many deterring secondary metabolites deposited in the cell wall (37–39). The cytoplasmic expression of nematotoxic proteins in fungi could therefore be a prime mechanism to defend against this type of predation. The fungivorous nematodes Aphelenchus avenae Bastian (40) and Bursaphelenchus okinawaensis strain SH1 (41) were used as model predators due to their ubiquitous presence in soils of temperate regions, where they cohabit in this ecosystem with most of the fungal species chosen for this study. These nematodes feed on the mycelium and fruiting body tissue, making them ideal candidate organisms to evaluate the toxicity of cytoplasmically expressed fruiting body defense proteins (FBDPs) (42, 43). Six different FBDPs belonging to the two FBDP classes introduced above were chosen for this study. We heterologously expressed these FBDPs individually in the cytoplasm of vegetative hyphae of the ascomycete Ashbya gossypii, thereby retaining the physiological context of the proteins.

The applied experimental system allowed a direct comparison of toxicity between the individual proteins. The observed susceptibility of a fungal predator to different FBDPs supports the hypothesis that these proteins are produced to confer fruiting body resistance to predation. Similar toxicities against fungivorous and bacterivorous nematodes were found, suggesting that the respective targets of the toxins are conserved between different species/classes of nematodes.

## RESULTS

**FIG 1:**
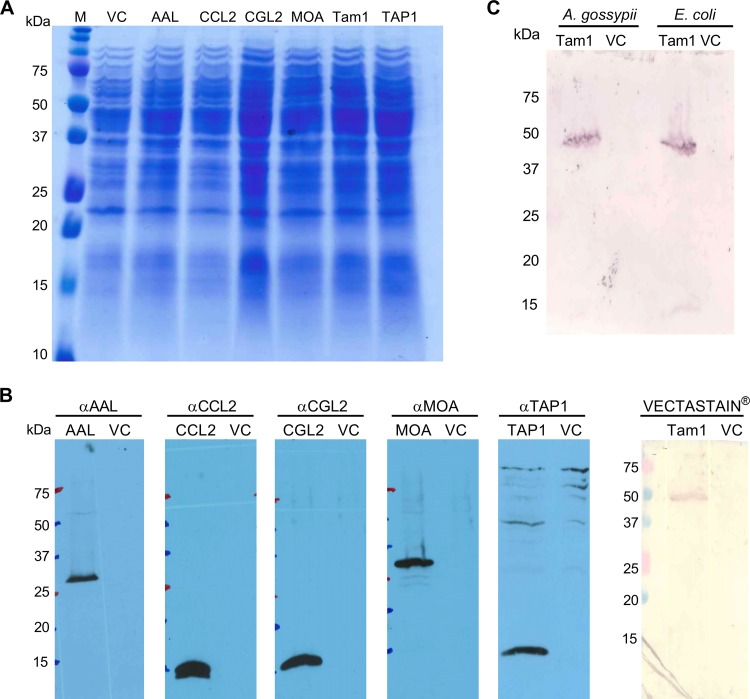
Expression analysis of A. gossypii transformants expressing different FBDPs. (A) Fungal lysate (20 μl) was loaded on an SDS-PAGE gel and stained with Coomassie brilliant blue. (B) Whole-cell protein extracts of A. gossypii transformants carrying the A. gossypii VC (VC) or one of the FBDP-encoding plasmids were analyzed by immunoblotting using FBDP-specific polyclonal antibodies. For detection of tamavidin 1, the Vectastain ABC alkaline phosphatase system was used. The expected molecular mass of each protein is given in Table 1. (C) Expression of tamavidin 1 in A. gossypii and E. coli was detected using the Vectastain ABC alkaline phosphatase system. The sizes of the marker proteins are indicated.

We selected a panel of six previously characterized FBDPs, covering a wide spectrum of fungal species, protein folds, and biochemical activities, to assess the biotoxicity of such proteins against the fungivorous nematodes A. avenae and B. okinawaensis (Table 1). The ascomycete A. gossypii was the expression host system of choice, because it can be easily modified genetically and expresses no orthologues of the FBDPs tested in this study. To obtain comparable results in a well-defined system and to maintain the physiological localization of the FBDPs during predation, we cloned and expressed each of the fungal FBDPs individually in the cytoplasm of A. gossypii and probed their expression (Fig. 1A). In immunoblot analyses, we could detect all proteins at their calculated molecular masses, except for Tam1, which was detected via its tetrameric, biotin-bound form (see Materials and Methods) (Fig. 1B). This detection method for Tam1 was validated by assaying the heterologous expression of the protein in Escherichia coli (Fig. 1C).

**TABLE 1:** Overview of the fruiting body defense proteins (FBDPs) tested for toxicity against A. avenae and B. okinawaensis^*a*^

Lectin/defense protein (FBDP)^*b*^	Molecular mass (kDa)	Origin	Protein family	Ligand specificity	Toxicity against:		GenBank accession no.	References
					A. aegypti	C. elegans		
CGL2	16.7	Coprinopsis cinerea	Galectin	Galβ(1,4)Glc, Galβ(1,4)GlcNAc, Galβ(1,4)Fuc	Toxic	Toxic	AAF34732	28, 48, 56
CCL2	15.3	Coprinopsis cinerea	Ricin B-type lectin	GlcNAcβ(1,4)[Fucα(1,3)]GlcNAc	Nontoxic	Toxic	EU659856	18, 57
TAP1	16.1	Sordaria macrospora	Actinoporin-like lectin	Galβ(1,3)GalNAc	Toxic	Toxic	CAH03681	23, 28
MOA	32.3	Marasmius *oreades*	Chimeric ricin B-type lectin	Galα(1,3)Gal	Toxic	Toxic	AY066013	35, 58
AAL	33.4	Aleuria aurantia	β-Propeller lectin	Fucose	Toxic	Toxic	BAA12871	28, 55, 59, 60
Tam1	15.1	Pleurotus cornucopiae	Biotin-binding protein	Biotin	Nontoxic	Toxic	AB102784	33, 46

a Six different FBDPs from five fungal species were cloned and expressed in A. gossypii in order to test their toxicity toward fungal-feeding nematodes A. avenae and B. okinawaensis. The six selected FBDPs were previously shown to be toxic to at least one of the indicated bacterivorous or omnivorous model organisms.

b CGL2, Coprinopsis cinerea galectin 2; CCL2, Coprinopsis cinerea lectin 2; AAL, Aleuria aurantia lectin; MOA, Marasmius *oreades* agglutinin; TAP1, Sordaria macrospora transcript associated with perithecial development 1; Tam1, tamavidin 1.

**FIG 2:**
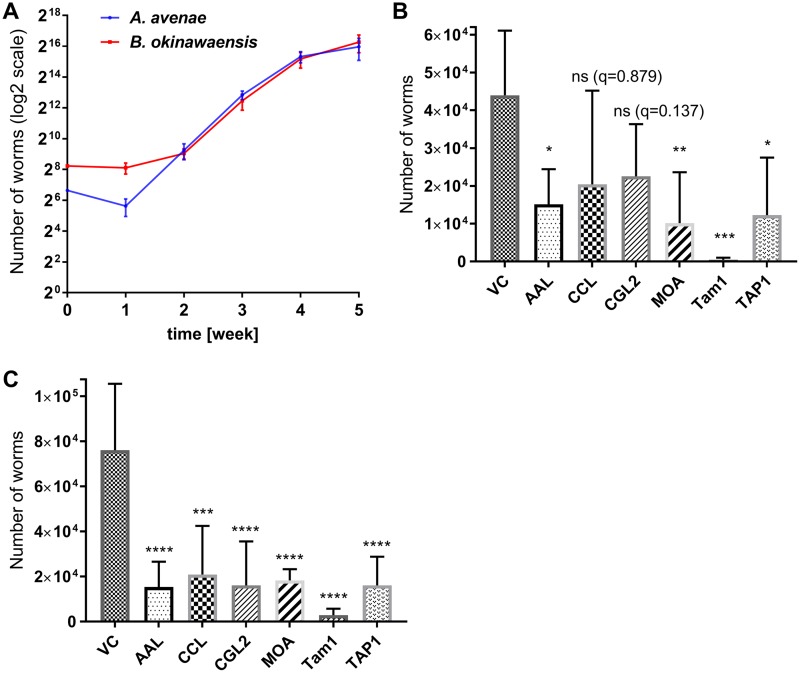
Propagation rate of fungivorous nematodes on different A. gossypii transformants. (A) A. avenae (100) or B. okinawaensis (300) nematodes were propagated on A. gossypii VC. Nematodes were harvested and counted after the indicated times of incubation. (B) Indicated FBDPs were individually expressed in the vegetative mycelium of A. gossypii. A. avenae (100) nematodes were inoculated on individual A. gossypii transformants and incubated for 28 days at 20°C. Thereafter, nematodes were harvested and counted. (C) Indicated FBDPs were individually expressed in the vegetative mycelium of A. gossypii. A total of 300 B. okinawaensis nematodes were inoculated on individual A. gossypii transformants and incubated for 28 days at 20°C. After this period, nematodes were harvested and counted. Each error bar represents the standard deviation of five biological replicates. Dunnett's multiple-comparison test was used for statistical analysis. ns, not significant; *, *P* < 0.05; **, *P* < 0.01, ***, *P* < 0.001; ****, *P* < 0.0001. Significance was determined versus VC.

In the propagation rate assay, both nematodes grew at an exponential rate on A. gossypii colonies, proving that it is a suitable food source for the fungivorous nematodes used in this study (Fig. 2A). In the case of B. okinawaensis, three times more nematodes were added to compensate for the slow growth of this worm compared to that of A. avenae on A. gossypii plates (Fig. 2A).

**FIG 3:**
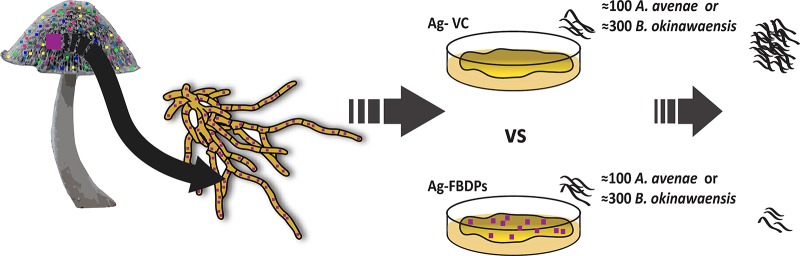
Schematic representation of the experimental setup. FBDPs from different fungal species were selected and individually expressed in A. gossypii vegetative mycelium. The indicated numbers of each nematode were picked and placed onto an A. gossypii colony harboring a control plasmid or expressing an FBDP. After 4 weeks, the coculture was harvested and nematodes were counted.

The toxicity of the individual FBDPs to fungivorous nematodes was assessed by comparing propagation of the nematodes on A. gossypii transformants, constitutively expressing one of the defense proteins, relative to that of an A. gossypii vector control (VC) strain (Fig. 3). After 28 days of cocultivation, the cultures were harvested and the sizes of the nematode populations were assessed. This period corresponds, according to the determined propagation rates, to the exponential growth phase of the nematodes (Fig. 2A) We found that four out of six tested FBDPs expressed in A. gossypii (Aleuria aurantia lectin [AAL], Marasmius oreades agglutinin [MOA], Sordaria macrospora transcript associated with perithecial development 1 [TAP1], and Tam1) conferred a significant inhibition of propagation for both nematodes (Fig. 2B and C). The two fruiting body lectins from Coprinopsis cinerea, Coprinopsis cinerea galectin 2 (CGL2) and Coprinopsis cinerea lectin 2 (CCL2), exhibited significant toxicity for B. okinawaensis, whereas the effect on the propagation of A. avenae was not statistically significant and was highly variable (Fig. 2B and C). Determination of the propagation rate of A. avenae nematodes fed with A. gossypii transformants expressing these two lectins was repeated with more biological replicates; however, the high variability among biological replicates was reproducible.

Three of the toxic FBDPs were lectins (Fig. 2B): AAL, a fucose-binding lectin from Aleuria aurantia; MOA, a chimerolectin expressed in the fruiting bodies of Marasmius oreades; and TAP1, an actinoporin-like lectin from perithecia of Sordaria macrospora. These lectins significantly slowed both A. avenae and B. okinawaensis propagation when constitutively expressed in A. gossypii. In these three treatments, the nematode populations were approximately a quarter of the size of that in the control. The most dramatic effect on nematode development was observed for treatments expressing the biotin-binding protein tamavidin 1 (Fig. 2B and C). When this protein was expressed in A. gossypii, very few nematodes (less than 4% compared to the VC strain population) were retrieved from the cocultures, indicating that expression of biotin-binding proteins strongly prevented the propagation of the fungivorous nematodes.

The toxicity of the tested FBDPs is similar to what was observed for the bacterivorous model organism C. elegans (Table 1), suggesting that the underlying target(s) is conserved among different nematode species.

## DISCUSSION

Fungal fruiting and resting (sclerotia) bodies express multiple defense toxins against predators (17, 44). The functional redundancy of these molecules with regard to toxicity makes it difficult to study the contribution of an individual compound or protein to fungal resistance towards a specific predator. Therefore, a synthetic approach is favored over the deletion of individual genes. We present here a heterologous fungal expression system that is similar to the physiological situation in the originating fungi and thus allows investigation of the role of an individual protein in fungal resistance towards fungivores.

The filamentous yeast A. gossypii appears to be an ideal tool for studying individual FBDPs and their contribution to the protein-mediated defense against a particular predatory species. The organism lacks orthologs of many FBDPs, possibly due to its phylogenetic relatedness to the yeast Saccharomyces cerevisiae and its adaptation to insects as an ecological niche (45), but it is readily used as prey by the model fungivorous nematodes (Fig. 2A). Expression analysis showed that all proteins are expressed and can be detected in A. gossypii (Fig. 1B). Due to the use of different antisera for the different FBDPs, no real quantitative statements about the expression levels of the various FBDPs can be made, however. The detection signal for tamavidin 1 was very weak at the expected molecular mass (15.1 kDa) of the monomer and much stronger at 50 kDa (Fig. 1B and C). Previously, it was shown that tamavidin 1 is a homotetramer in its native form. The molecular mass of the homotetramer was estimated to be around 50 kDa, based on gel filtration chromatography (46), which agrees with our findings. We hypothesize that the denaturing conditions of the SDS-PAGE lead to dissociation of most of the Tam1 homotetramers into monomers, but since the native tetrameric form binds biotin with high affinity, it is better detected with an avidin-based detection reagent. Resistance of homotetrameric Tam1 to SDS-PAGE denaturing conditions was confirmed by expressing the protein in E. coli (Fig. 1C).

The biotin-binding protein tamavidin 1 was highly effective against both A. avenae and B. okinawaensis (Fig. 2B and C). Toxicity of this protein is thought to depend on the sequestration of free biotin, thus reducing the bioavailability for the predator of this essential nutrient (29, 33, 47). The sequestration of an essential vitamin by a proteolysis-resistant, high-affinity binding protein seems to be a powerful way to prevent predation. Our results confirm those of previous studies, in which biotin-binding proteins have been implicated in fungal resistance and employed as effective repellents in plants for several different predators and parasites (33, 47). The lack of toxicity of tamavidin 1 expression for the producing organisms E. coli (33) and A. gossypii (this study) suggests that there is no freely available biotin in the cytoplasm of these organisms.

Besides tamavidin 1, three of the tested lectins, AAL, MOA, and TAP1, were clearly toxic for both of the nematodes (Fig. 2B and C). In comparison to the biotin-binding protein, however, growth was not dramatically abolished, and the extent to which the lectins attenuated population growth varied. The C. cinerea fruiting body lectins CGL2 and CCL2 showed inconsistent effects on the A. avenae population size after 28 days, even though they were shown to be strongly toxic to the model nematode C. elegans (18, 28). The variable toxicity of these proteins toward A. avenae may be explained by the existence of specific mechanisms of this nematode to escape intoxication by these toxins, e.g., through changes of the glycans in the digestive tract. Thus far, there is no report about such a mechanism, but it has been shown in C. elegans that mutations in specific glycosyltransferases can confer resistance to both CGL2 and CCL2 (18, 48). The lower nematotoxicity of these proteins expressed in the A. gossypii (Fig. 1A) compared to E. coli (18, 28, 48) may be due to the lower expression levels of CGL2 and CCL2 in the fungus compared to those in the bacterium.

With this study, we present experimental evidence that FBDPs are involved in the defense of fungi against fungivores. Based on our experiments, we cannot make a statement, however, about how the observed toxicity would protect the fungus from predation. Based on previous experiments with FBDP-expressing E. coli and C. elegans, we hypothesize that the nematodes would avoid feeding on the toxic fungus (28). The heterologous expression system for FBDPs implemented here allows the comparison of many different candidate proteins and protein classes for their toxicity against the fungivorous nematodes A. avenae and B. okinawaensis and other fungivores. Since some of these proteins are abundant in edible mushrooms, and A. avenae also feeds on plant epidermal cells and root hairs (38), our results open possible avenues for crop protection, e.g., by expression of FBDPs from edible mushrooms in crops.

## MATERIALS AND METHODS

### Strains and cultivation conditions.

Escherichia coli strains DH5α and BL21 were used for cloning and protein purification, respectively. Both strains were cultivated on LB medium, as described previously (49). The nematodes A. avenae (Bastian, 1865) and B. okinawaensis (strain SH1) were maintained on a sporulation-deficient strain (BC-3) of Botrytis cinerea cultivated on malt extract agar medium (MEA) supplemented with an additional 15 g/liter agar and 100 μg/ml chloramphenicol at 20°C in the dark (41, 50). Nematodes were extracted from cocultures by Baermann funneling (51, 52) and decontaminated on 1.5% water-agar plates containing 200 μM G418 (Geneticin) and 50 μg/ml kanamycin for 2 days at 20°C (28). Toxicity assays were performed with A. gossypii ATCC 10895 and its transformants created in this study (Table 2) on solid Ashbya full medium (AFM) (1% [wt/vol] yeast extract, 1% [wt/vol] peptone, 1.5% [wt/vol] agar, 2% [wt/vol] glucose, 0.1% [wt/vol] myo-inositol, and 200 μM G418) at 20°C (53).

**TABLE 2:** A. gossypii plasmids and strains generated and used for this study

Plasmid	Markers	Insert	Source or reference	Resulting strain
pRS-AgTEFp-GFP	Amp^r^, *GEN3*	GFP^*a*^	61	*Ag*GFP
pRS-AgTEF-VC	Amp^r^, *GEN3*	None	This study	*Ag*VC
pRS-AgTEF-CGL-2	Amp^r^, *GEN3*	CGL2	This study	*Ag*CGL2
pRS-AgTEF-CCL-2	Amp^r^, *GEN3*	CCL2	This study	*Ag*CCL2
pRS-AgTEF-TAP-1	Amp^r^, *GEN3*	TAP1	This study	*Ag*TAP-1
pRS-AgTEF-MOA	Amp^r^, *GEN3*	MOA	This study	*Ag*MOA
pRS-AgTEF-AAL	Amp^r^, *GEN3*	AAL	This study	*Ag*AAL
pRS-AgTEF-Tam1	Amp^r^, *GEN3*	Tam1	This study	*Ag*Tam1

a GFP, green fluorescent protein.

### Cloning and expression of fungal lectins and other cytoplasmic defense proteins in A. gossypii.

We selected a total of six previously characterized fruiting body-specific lectins and a biotin-binding protein from five different fungal species (Table 1). The primers and plasmids used to amplify the respective cDNAs from pET expression vectors, as well as the plasmids generated in this study, are listed in Tables 3 and 2, respectively.

**TABLE 3:** Primer sequences used for amplification of FBDP-coding sequences and their cloning into A. gossypii expression vector pRS-AgTEF

Primer	Sequence (5′ to 3′)	Parental plasmid	Reference
CGL2 fwd SalI	GGGGGGGTCGACATGCTCTACCACCTTTTCGTCAAC	pET24b-CGL2	62
CGL2 rev AscI	GGGGGGGGCGCGCCCTAAGCAGGGGGAAGTGGG		
CCL2 fwd SalI	GGGGGGGTCGACATGGACTCCCCAGCTGTGAC	pET24b-CCL2	18
CCL2 rev AscI	GGGGGGGGCGCGCCCTAGACCTTCTCGATGACCC		
TAP1 fwd XhoI	AAAAAACTCGAGGTCGACATGTCCTACACCCTCCACCTCCGT	pET24b-TAP1	28
TAP1 rev AscI	AAAAAAGGCGCGCCTCAAAGATACTCAACCGTAGCCCT		
MOA fwd SalI	GATGTCGTCGACCATATGTCTCTGCGACGCGGAATTTAC	pET22-MOA	35
MOA rev AscI	GTATTAGGCGCGCCCTCAGTAGAAGGCCATGTAGCTGTC		
AAL fwd SalI	GGGGGGGTCGACATGCCTACCGAATTCCTCTAC	pET28b-AAL	28
AAL rev AscI	GGGGGCGCGCCTTACCATCCCGCGGGAGTG		
Tam1 fwd SalI	TTTTTTGTCGACATGAAAGACGTCCAATCTCTCCTCACC	pET24b-Tam1	33
Tam1 rev AscI	TTTTTTGGCGCGCCTCACTCGAACTTCAACCCGCGACG		

As a general cloning strategy, the green fluorescent protein (GFP)-encoding sequence in the A. gossypii expression plasmid pRS-*Ag*TEFp-GFP was replaced by FBDP-encoding sequences using the restriction sites SalI/XhoI and AscI. The sequences of the resulting plasmids were verified by Sanger sequencing. Plasmids were transformed into A. gossypii by electroporation as described previously (54). Transformants were selected on AFM plates containing 200 μM G418. Homokaryons were produced by a second selection of spores isolated from transformants on AFM plates containing 200 μM G418.

### Protein expression analysis in A. gossypii.

A. gossypii transformants were grown on AFM containing 200 μM G418 at 28°C for 7 days. Protein expression was verified by harvesting the mycelium and preparing whole-cell protein extracts of the A. gossypii transformant colonies, as described previously (28). The prepared extracts were analyzed by immunoblotting using specific antisera (1:2,500 dilutions) for detection of the various FBDPs, except for the extracts containing tamavidin 1, where the blotted extract proteins were probed for bound biotin using the Vectastain ABC alkaline phosphatase system (Vector Laboratories) in combination with a 1-Step NBT/BCIP (nitro-blue tetrazolium/5-bromo-4-chloro-3′-indolyl phosphate) solution (Thermo Scientific). Antisera against Coprinopsis cinerea galectin 2 (CGL2) and Coprinopsis cinerea lectin 2 (CCL2) were described previously (18, 28). The rabbit antisera against Marasmius oreades agglutinin (MOA), Aleuria aurantia lectin (AAL) and Sordaria macrospora transcript associated with perithecial development 1 (TAP1) were raised against the purified recombinant proteins by Pineda Antikörper-Service (Berlin, Germany). Expression and purification of these proteins from E. coli were carried out as previously described (28, 35, 55).

### Determination of propagation rates of fungivorous nematodes on A. gossypii.

Propagation rates of A. avenae and B. okinawaensis nematodes on A. gossypii were determined by transferring 100 and 300 mixed-stage worms, respectively, onto a 7-day-old A. gossypii VC colony harboring pRS-AgTEF-VC (Table 2) on AFM plates containing 200 μM G418. During subsequent incubation of the cocultures for 35 days at 20°C, nematodes from five plates were harvested in parallel every week by Baermann funneling overnight at room temperature. The funneled volume was adjusted to 0.5 to 30 ml based on the estimated number of nematodes and counted six times by taking different aliquots each time. The total number of nematodes extracted from each plate was determined by multiplying the average of the six counts by the appropriate conversion factor (see Supplemental File 1). Thus, for each time point, five biological replicates were analyzed.

### Toxicity assays of FBDPs toward fungivorous nematodes.

The toxicities of the individual toxins against fungivorous nematodes were assessed by cultivating A. gossypii transformants expressing the various FBDPs or carrying the expression vector control (VC) on AFM plates containing 200 μM G418 for 7 days at 28°C before inoculating the plates with 100 or 300 mixed-stage A. avenae or B. okinawaensis nematodes, respectively. The cocultivation plates were incubated at 20°C for 4 weeks. Subsequently, the plates were harvested by overnight Baermann funneling. The nematode population was assessed as described above. All assays were performed with five biological replicates. The individual counts and results of each biological replicate are listed in Supplemental File 1. Each data point in Fig. 2A, B, and C corresponds to the mean of five biological replicates.

### Statistical analysis.

The statistical significance of the difference between the means of the nematode abundances for the various FBDPs and those of the respective controls was assessed using one-way analysis of variance followed by Dunnett's multiple-comparison test. All statistical analyses were conducted using GraphPad Prism 7 (GraphPad, San Diego, CA).

## Supplementary Material

Supplemental file 1
